# Community health workers augment the cascade of TB detection to care in urban slums of two metro cities in India

**DOI:** 10.7189/jogh.11.04042

**Published:** 2021-07-17

**Authors:** Rajaram Subramanian Potty, Karthikeyan Kumarasamy, Rajesham Adepu, Ramesh Chandra Reddy, Anil Singarajipura, Poornima Bathi Siddappa, Prarthana B Sreenivasa, Raghavendra Thalinja, Mohan Harnahalli Lakkappa, Reuben Swamickan, Amar Shah, Vikas Panibatla, Ramesh Dasari, Reynold Washington

**Affiliations:** 1 Karnataka Health Promotion Trust, Bengaluru, Karnataka, India; 2 Office of the Joint Director (TB), Commissionerate of Health and Family Welfare, Hyderabad, Telangana, India; 3 Office of the Joint Director (TB), Lady Willingdon State TB Centre, Bengaluru, Karnataka, India; 4 Tuberculosis and Infectious Diseases Division, USAID/India, New Delhi, India; 5 TB Alert India, Hyderabad, India; 6 Department of Community Health Sciences, University of Manitoba, Winnipeg, Manitoba, Canada; 7 St John’s Research Institute, Bengaluru, India

## Abstract

**Background:**

Tuberculosis Health Action Learning Initiative (THALI) funded by USAID is a person-centered initiative, supporting vulnerable urban populations to gain access to TB services. THALI trained and placed 112 Community health workers (CHWs) to detect and support individuals with TB symptoms or disease within urban slums in two cities, Hyderabad and Bengaluru, covering a population of about 3 million.

**Methods:**

CHWs visited the slums once in a fortnight. They conducted TB awareness activities. They referred individuals with TB symptoms for sputum testing to nearest public sector laboratories. They visited those testing TB positive, once a fortnight in the intensive phase, and once a month thereafter. They supported TB patients and families with counselling, contact screening and social scheme linkages. They complemented the shortfall in urban TB government field staff numbers and their capacity to engage with TB patients. Data on CHWs’ patient referral for TB diagnosis and treatment support activities was entered into a database and analyzed to examine CHWs’ role in the cascade of TB care. We compared achievements of six monthly referral cohorts from September 2016 to February 2019.

**Results:**

Overall, 31 617 (approximately 1%) of slum population were identified as TB symptomatic and referred for diagnosis. Among the referred persons, 23 976 (76%) underwent testing of which 3841 (16%) were TB positive. Overall, 3812 (99%) were initiated on treatment and 2760 (72%) agreed for regular follow up by the CHWs. Fifty-seven percent of 2952 referred were tested in the first cohort, against 86% of 8315 in the last cohort. The annualized case detection rate through CHW referrals in Bengaluru increased from 5.5 to 52.0 per 100 000 during the period, while in Hyderabad it was 35.4 initially and increased up to 118.9 per 100 000 persons. The treatment success rate was 87.1% among 193 in the first cohort vs 91.3% among 677 in the last cohort.

**Conclusions:**

CHWs in urban slums augment TB detection to care cascade. Their performance and TB treatment outcomes improve over time. It would be important to examine the cost per TB case detected and successfully treated.

India continues to contribute greatly to the global tuberculosis (TB) burden due to its large population size and high TB incidence. The Global TB report 2020 indicated that India was listed as one of 30 high TB burden countries and accounted for 26% of the TB cases globally [1]. India also accounts for 25% of total estimated missing cases globally, a large gap between the estimated and reported number of new TB cases [2]. Many factors contribute to this scenario. About half of TB symptomatic persons first seek care within the private sector which is not yet fully engaged in TB control [3]. Under-reporting of TB patients as a result of system constraints or attitudes of health personnel are other reasons for this gap [4-11]. In order to close this gap in TB detection, intensified efforts are required especially among populations who are more vulnerable to TB and who have challenges in accessing TB diagnostic services [2]. Vulnerable populations with TB symptoms need knowledge and access to TB diagnostic and treatment facilities in order to detect the disease in a timely manner.

Early diagnosis and treatment for TB reduces the risk of disease transmission from one individual to others in the family or in close contact [12]. Early initiation of treatment also improves TB treatment outcomes and reduces the complications as well as the out of pocket expenditure incurred by individuals and their families [13,14]. The two well-known pathways to early diagnosis of TB are the patient-initiated pathway and the screening-pathway [15]. In the patient-initiated pathway, the person with TB actively seeks care from the health care provider when he or she experiences symptoms. In the screening pathway, individuals are screened for symptoms suggestive of TB and the persons with TB suggestive symptoms are referred for appropriate TB testing. The latter approach has the potential advantage of diagnosing TB in persons who would have otherwise missed or delayed their diagnosis [15]. However, in order to be cost-efficient with this approach, the focus must be among populations that are at a greater risk of developing TB disease. Among others, these include individuals living in urban slums, individuals who are exposed to occupational environments that compromise lung function, individuals who are severely malnourished, and persons living with HIV [16-20].

The United States Agency for International Development/India (USAID/India) awarded a grant namely ‘Tuberculosis Health Action Learning Initiative (THALI)’ to a consortium of partners led by Karnataka Health Promotion Trust (KHPT), Bengaluru. KHPT works in close partnership with TB Alert India (TBAI) in Hyderabad, and in collaboration with the Revised National Tuberculosis Control program (RNTCP) now referred to as the National TB Elimination Program (NTEP). The THALI project is a patient centered, family-focused TB prevention and care initiative supporting vulnerable people to gain access to quality TB care services from health care providers of the patient’s choice. One of the strategies adopted by THALI was to enhance community outreach and engagement within vulnerable communities through a dedicated cadre of community health workers (CHWs). This was necessary, considering the 30%-50% gap in recruitment of TB health visitors (TBHV) in metro-cities and large towns, the front-line work force within the NTEP. As well, the TB case load within these high TB prevalent communities’ demands additions to the work-force in order to be able to support TB patients with counselling for treatment adherence and linkages to nutrition and other social support schemes, that often remain inaccessible to these populations. Shortfalls in the number and capacity of front-line health care workers weakens the last mile in the delivery of TB care services. Innovative strategies such as development of alternative cadres and task shifting are attempts to address these shortfalls [20,21]. A systematic analysis of the program learnings from community engagement activities that are conducted through CHWs is important to contribute to evidence-based policy and program related decision-making [22]. In this paper we systematically analyze and describe the potential augmentation in the TB care cascade achieved through the community engagement activities conducted in two large metro cities in India, namely Bengaluru and Hyderabad. The uniqueness of this paper is the depiction of the entire continuum of care of TB patients through community engagement.

## METHODS

### Study setting, design and data collection

The project area included urban slum areas of Hyderabad and Bengaluru cities. We identified and mapped 647 slums in Bengaluru city and 942 slums in Hyderabad city, catering to about 323 000 households and 1.5 million population covered by 24 TB units (TUs) in Bengaluru and about 294 000 households and 1.45 million population covered by 19 TUs in Hyderabad.

The THALI project trained and placed 112 CHWs to support individuals with TB symptoms or confirmed disease living within urban slums in the two cities, Hyderabad and Bengaluru. Each CHW covered about 5-25 slum areas with a population ranging from 20 000-32 000 and aligned to the geographic coverage by the Designated Microscopy Centre (DMC), a government health care facility that conducts sputum microscopy and onward referral for Cartridge Based Nucleic Acid Amplification Test (CBNAAT) for TB diagnosis. Fourteen Community Coordinators (CC) supervised and supported about 8-10 CHWs, each. CHWs conducted Information Education and Communication (IEC) campaigns on TB within the community through in-person contacts, small group meetings, large group meetings and school-based education programs and were involved in the periodic active case finding campaigns that were conducted by the government. CHWs visited each slum area once in a fortnight.

Using a ‘screening pathway’, CHWs actively identified individuals with TB suggestive symptoms during the community engagement activities listed above. Any person who had persistent cough for more than 14 days and/or had night sweats, sudden weight loss, blood in sputum, reduced appetite, persistent chest pain and enlarged lumps (lymph nodes) was identified as a TB symptomatic. They referred TB symptomatics for sputum testing to the DMC, a public health facility for TB diagnosis. The referral process included collecting socio-demographic details, filling up a form in triplicate and handing over two sputum cups, with clear instructions on how to collect an on-the-spot sample and an early morning sputum sample. If the individual could not go themselves to the DMC to hand over the samples for testing, the CHW would transport the sample for testing on behalf of the referred person, adhering to standard infection prevention guidelines that they were trained in. Whenever a sample tested positive for TB, the CHW would accompany that individual to the public health facility for counselling and treatment initiation. Following initiation of the TB treatment, with verbal recorded consent of the individual, the CHW followed up the person twice a month during the intensive phase and once a month during the continuation phase of treatment. During the follow-up visits, the CHW would mobilize family level support, provide counselling support and monitor treatment adherence, give nutritional advice and support the individual to obtain social entitlements, including enrolment into the Government’s direct benefit transfer (DBT) scheme of Indian rupees 500 per month. Often, these patients were supported to open a bank account and link their Aadhar (Unique Identification Number) and telephone number to the bank account, in order to avail the DBT. Many patients were not readily willing to share these details, as they feared misuse of the data. TB patients and family level treatment supporters were also motivated to attend patient support group (PSG) meetings. The patient’s weight gain was monitored during follow-up visits. Patients were reminded about and referred for follow-up tests, counselled on relevant behavior change (smoking and alcohol consumption) and referred to a doctor for management of adverse drug reactions or for side effects management and co-morbidities.

**Figure 1:**
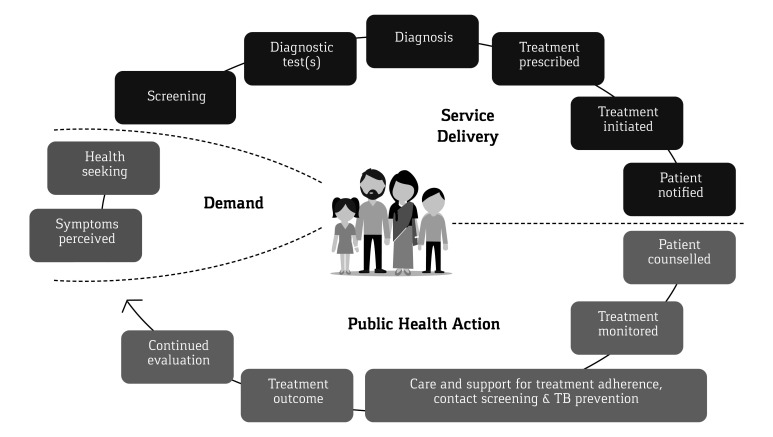
The continuum of tuberculosis care adopted by the THALI CHW.

All counselling inputs by the CHW, weight measurements, follow-up test results and treatment adherence were documented by the CHW, under the supportive supervision of the CC, on a Patient Referral and Diagnosis (PRAD) card form during referral and a Prevention, Care and Support card (PCS) during treatment adherence support. The outcome of the TB treatment was recorded and validated by project medical personnel and the NTEP Senior TB Treatment Supervisor (STS). Both forms once filled were verified for completeness by the CC, before entry into the computerized management information system (CMIS) on a regular basis. The continuum of TB care cascade considered in the present analysis starts from the point when the referrals were made and ends with the treatment outcome. We did not collect any data on incidents or events pertaining to these individuals prior to their referrals. **Figure 1** provides the visual image of the continuum of TB care cascade adopted in THALI.

### Data analysis

We analyzed both the PRAD and the PCS data collected by the CHW. In order to examine the trends in the TB care cascade, we grouped the referred patients into various cohorts according to the referral months and year. We made five cohorts each of six months’ duration based on the months and year of referral, starting in September 1, 2016 and ending as of February 28, 2019. We examined the number of referrals, the proportion of persons who had undergone the tests, the proportion among them diagnosed as TB either microbiologically or clinically, and proportion started on anti-tuberculosis treatment (ATT). For each of these cohorts, we examined the percentage of patients with successful treatment outcomes, that is who are either declared cured of TB or have completed the course of TB treatment. In addition, we also examined successful treatment outcomes according to socio-economic and demographic characteristics, number of follow-up visits, whether missed any dose during the treatment, and type of action taken by the CHW during the follow-up visit. We applied the multiple logistic regression model to identify the likelihood of successful treatment outcome across the referral cohorts. We adopted stepwise backward logistic regression models to explore significant changes in the successful treatment outcomes over the time periods. If the *P*-value of any group as a whole was more than 0.10, such variables were eliminated from the multiple logistic regression. We constructed three different logistic regression models that included the number of follow-up visits during the intensive phase, the number of follow-up visits during the continuation phase and the total number of follow-up visits in addition to the various characteristics mentioned. We applied three different models for these three visits separately in order to understand the overall change in the treatment outcome across various referral cohorts, after controlling for these visits along with other potential factors. It is also possible that these visits can be potentially interrelated and we thus chose not to use these three visits in the same model. The data analysis was conducted using Stata 14.2 SE (Stata Corp, College Station, TX, USA).

The community-based activities through the CHWs aimed to increase TB case finding, and to improve TB treatment outcomes. Numerous research studies conducted locally and in other countries have documented several socio-demographic, clinical, and treatment characteristic of the patient associated with TB treatment outcomes. In general, the various potential risk factors considered and documented to be associated with TB treatment outcomes in the previous researches include age, gender, type of TB, comorbidity status, history of previous treatment status, counselling during the treatment, treatment adherence, number of follow-up visits, literacy status, religion or ethnicity, distance to care center and nutritional status of the patient [23-32]. The results from these research studies differed according to the setting and study population. In the present study we used the potential risk factors identified by previous research and the availability of information in the PCS and PRAD data.

### Ethical approval

The Institutional Ethics Committee of St John’s Medical College and Hospital provided the ethics approval for program data review and analysis. The State TB office and local NTEP officials in the two states provided regulatory approval for access to Nikshay data and to interview patients and conduct follow-up visits.

## RESULTS

Overall, from September 2016 to February 2019, the CHWs in Bengaluru and Hyderabad had referred 31 617 TB symptomatic individuals for TB diagnoses (approximately 1% of the total slum population). Of those referred, 23 976 (76%) persons had completed TB testing and were provided with results (**Table 1**). The proportion of referred individuals undergoing testing increased over time from 57% in the first cohort to 86% in the last. This increase to over 80% of those referred being tested and receiving results was seen in both cities, across gender and all age groups. The annualized case detection rate through CHW referrals in Bengaluru increased from 5.5 to 52.0 per 100 000 persons during the period, while in Hyderabad it was 35.4 initially and increased up to 118.9 per 100 000 persons.

**Table 1:** Coverage indicators reflecting the cascade of TB care including number of persons referred by CHW, undergone test, tested positive, started treatment, followed-up according to various referral cohorts

Six monthly referral cohorts	Number referred	Number tested	Number tested positive	Number started treatment	Registered for follow-up by CHW	% undergone test	% tested positive	% started treatment	% Registered for follow-up
**Total:**									
Sep 2016-Feb 2017	2952	1691	298	296	203	57.3	17.6	99.3	68.6
Mar-Aug 2017	4176	2525	516	515	362	60.5	20.4	99.8	70.3
Sep 2017-Feb 2018	8270	6077	1082	1079	800	73.5	17.8	99.7	74.1
Mar-Aug 2018	7904	6521	1022	1010	696	82.5	15.7	98.8	68.9
Sep 2018-Feb 2019	8315	7162	923	912	699	86.1	12.9	98.8	76.6
**Bengaluru:**									
Sep 2016 Feb 2017	1179	588	41	40	31	49.9	7.0	97.6	77.5
Mar-Aug 2017	1946	1118	105	104	85	57.5	9.4	99.0	81.7
Sep 2017-Feb 2018	3436	2632	220	217	185	76.6	8.4	98.6	85.3
Mar-Aug 2018	3593	3159	303	294	226	87.9	9.6	97.0	76.9
Sep 2018-Feb 2019	4978	3979	393	382	315	79.9	9.9	97.2	82.5
**Hyderabad:**									
Sep 2016-Feb 2017	1773	1103	257	256	172	62.2	23.3	99.6	67.2
Mar-Aug 2017	2230	1407	411	411	277	63.1	29.2	100.0	67.4
Sep 2017-Feb 2018	4834	3445	862	862	615	71.3	25.0	100.0	71.3
Mar-Aug 2018	4311	3362	719	716	470	78.0	21.4	99.6	65.6
Sep 2018-Feb 2019	3337	3183	530	530	384	95.4	16.7	100.0	72.5
**Male:**									
Sep 2016-Feb 2017	1549	893	170	169	114	57.7	19.0	99.4	67.5
Mar-Aug 2017	2142	1305	290	289	213	60.9	22.2	99.7	73.7
Sep 2017-Feb 2018	4159	3067	574	573	429	73.7	18.7	99.8	74.9
Mar-Aug 2018	4073	3361	534	524	360	82.5	15.9	98.1	68.7
Sep 2018-Feb 2019	4286	3674	513	504	387	85.7	14.0	98.2	76.8
**Female:**									
Sep 2016-Feb 2017	1403	798	128	127	89	56.9	16.0	99.2	70.1
Mar-Aug 2017	2034	1220	226	226	149	60.0	18.5	100.0	65.9
Sep 2017-Feb 2018	4111	3010	508	506	371	73.2	16.9	99.6	73.3
Mar-Aug 2018	3831	3160	488	486	336	82.5	15.4	99.6	69.1
Sep 2018-Feb 2019	3766	3488	410	408	312	92.6	11.8	99.5	76.5
**Age <15 years:**									
Sep 2016-Feb 2017	185	90	11	11	7	48.6	12.2	100.0	63.6
Mar-Aug 2017	334	174	24	24	19	52.1	13.8	100.0	79.2
Sep 2017-Feb 2018	833	539	55	55	43	64.7	10.2	100.0	78.2
Mar-Aug 2018	636	452	68	68	36	71.1	15.0	100.0	52.9
Sep 2018-Feb 2019	489	394	32	32	23	80.6	8.1	100.0	71.9
**Age 15-49 years:**									
Sep 2016-Feb 2017	1164	656	224	222	154	56.4	34.1	99.1	69.4
Mar-Aug 2017	2367	1388	391	390	263	58.6	28.2	99.7	67.4
Sep 2017-Feb 2018	4346	2972	795	793	580	68.4	26.7	99.7	73.1
Mar-Aug 2018	5410	4435	765	758	529	82.0	17.2	99.1	69.8
Sep 2018-Feb 2019	5576	4749	707	700	537	85.2	14.9	99.0	76.7
**Age 50+ years:**									
Sep 2016-Feb 2017	576	361	63	63	42	62.7	17.5	100.0	66.7
Mar-Aug 2017	888	500	101	101	80	56.3	20.2	100.0	79.2
Sep 2017-Feb 2018	1800	1309	232	231	177	72.7	17.7	99.6	76.6
Mar-Aug 2018	2083	1691	189	184	131	81.2	11.2	97.4	71.2
Sep 2018-Feb 2019	2299	1959	184	180	139	85.2	9.4	97.8	77.2
**Total**	**31 617**	**23 976**	**3841**	**3812**	**2760**	**75.8**	**16.0**	**99.2**	**72.4**

CHW – community health worker, TB – tuberculosis

Overall, 16% of those tested were diagnosed to have TB and the TB detection among those tested reduced from 18% to 13% across the referral cohorts examined. The proportion of people who tested TB positive was consistently higher in Hyderabad than in Bengaluru, among males than among females and among those aged 15-49.

Overall, 99% of individuals diagnosed with TB were started on ATT with minor fluctuations in levels between Bengaluru and Hyderabad. Overall, 72% of them consented to be registered and documented for the follow-up visits using the PCS card, with proportions ranging between 69% and 77% across the referral cohorts. The proportion of TB patients who consented to be registered for PCS is comparatively lower in Hyderabad as compared to Bengaluru for all the referral cohorts examined. Also, in few of the cohorts slightly lesser proportion of TB patients aged below 15 years were registered for follow-up visits as compared to TB patients in other age groups.

**Table 2** provides the mean and total number of follow-up visits during the intensive and the continued treatment phases. Though, fluctuations were observed in the mean number of follow-up visits with Bengaluru indicating a reduction and Hyderabad showing an increase across various referral cohorts, the standard deviation reduced across groups indicating that the intensity of follow up became more or less uniform over time. We also noticed the same kind of results for the visits in the intensive and continuation phases as well.

**Table 2:** Mean number of total follow-up visits, mean number of visits during intensive phase and during continuation phase for TB patients referred by CHW according to different referral cohorts

	Total follow-up visits		Follow-up visits at continuation phase		Follow-up visits at intensive phase		Number of cases
**Referral cohorts**	**Mean**	**SD**	**Mean**	**SD**	**Mean**	**SD**	
**Total:**							
Sep 2016-Feb 2017	6.2	4.2	3.8	2.8	2.4	2.0	203
Mar-Aug 2017	7.4	5.0	4.5	3.2	2.9	2.4	362
Sep 2017-Feb 2018	7.5	4.2	4.4	2.7	3.0	2.1	800
Mar-Aug 2018	6.9	2.9	3.8	2.3	3.1	1.3	696
Sep 2018-Feb 2019	8.4	3.6	5.0	3.1	3.4	1.2	699
**Bengaluru:**							
Sep 2016-Feb 2017	11.0	6.8	5.8	4.7	5.2	2.9	31
Mar-Aug 2017	12.5	7.0	6.8	4.8	5.6	2.9	85
Sep 2017-Feb 2018	11.4	6.1	6.0	4.2	5.4	2.7	185
Mar-Aug 2018	7.5	3.1	4.0	2.5	3.5	1.4	226
Sep 2018-Feb 2019	7.3	3.3	4.2	2.7	3.1	1.2	315
**Hyderabad:**							
Sep 2016-Feb 2017	5.3	2.7	3.4	2.1	1.9	1.3	172
Mar-Aug 2017	5.8	2.7	3.7	2.0	2.1	1.3	277
Sep 2017-Feb 2018	6.3	2.3	3.9	1.9	2.3	1.1	615
Mar-Aug 2018	6.7	2.7	3.8	2.2	2.9	1.2	470
Sep 2018-Feb 2019	9.3	3.6	5.7	3.2	3.6	1.2	384
**Male:**							
Sep 2016-Feb 2017	6.2	4.7	3.7	3.0	2.5	2.3	114
Mar-Aug 2017	8.0	5.4	4.8	3.4	3.1	2.5	213
Sep 2017-Feb 2018	7.9	4.5	4.6	3.0	3.2	2.2	429
Mar-Aug 2018	7.1	2.9	3.9	2.4	3.2	1.3	360
Sep 2018-Feb 2019	8.4	3.8	5.1	3.3	3.3	1.2	387
**Female:**							
Sep 2016-Feb 2017	6.1	3.4	3.9	2.5	2.2	1.6	89
Mar-Aug 2017	6.5	4.4	3.9	2.7	2.6	2.2	149
Sep 2017-Feb 2018	7.0	3.7	4.1	2.4	2.8	1.9	371
Mar-Aug 2018	6.8	2.8	3.8	2.2	3.0	1.3	335
Sep 2018-Feb 2019	8.4	3.4	4.9	2.8	3.5	1.2	312
**Age <15 years:**							
Sep 2016-Feb 2017	7.4	3.2	5.3	3.1	2.1	1.6	7
Mar-Aug 2017	6.1	4.0	3.9	2.6	2.2	1.8	19
Sep 2017-Feb 2018	6.6	3.8	4.0	2.5	2.6	1.8	43
Mar-Aug 2018	7.1	2.7	3.8	1.9	3.3	1.2	36
Sep 2018-Feb 2019	7.7	2.7	4.6	2.1	3.1	1.3	23
**Age 15-49 years:**							
Sep 2016-Feb 2017	6.3	4.3	3.9	2.8	2.4	2.1	154
Mar-Aug 2017	6.9	4.5	4.2	2.8	2.7	2.2	263
Sep 2017-Feb 2018	7.4	4.2	4.4	2.8	3.0	2.1	580
Mar-Aug 2018	7.0	2.8	3.9	2.3	3.1	1.3	529
Sep 2018-Feb 2019	8.4	3.7	5.0	3.1	3.4	1.2	537
**Age 50+ years:**							
Sep 2016-Feb 2017	5.4	3.7	3.2	2.5	2.2	1.9	42
Mar-Aug 2017	9.1	6.4	5.4	4.2	3.7	2.8	80
Sep 2017-Feb 2018	7.8	4.3	4.5	2.7	3.2	2.2	177
Mar-Aug 2018	6.6	3.0	3.6	2.5	3.0	1.2	131
Sep 2018-Feb 2019	8.4	3.7	5.1	3.1	3.3	1.2	139
**Total**	7.5	3.9	4.4	2.8	3.1	1.8	2760

CHW – community health worker, TB – tuberculosis, SD – standard deviation

**Table 3** provides the percentage of TB patients having successful treatment outcomes according to the various referral cohorts. Overall, we noticed that the percentage of patients who experienced successful treatment outcomes improved over time from 87.1% to 91.3%, with small fluctuations in between. The increase in successful treatment outcomes was highest in Bengaluru and among those aged >50 years of age. The successful treatment outcomes were consistently higher (>90%) among females than among males for all the cohorts examined.

**Table 3:** Successful treatment according to various referral cohorts of patients who are referred and followed-up by the CHW*

Referral cohorts	% with successful outcomes	Number of cases
**Total:**		
Sep 2016-Feb 2017	87.1	193
Mar-Aug 2017	90.3	339
Sep 2017-Feb 2018	89.0	735
Mar-Aug 2018	90.1	636
Sep 2018-Feb 2019	91.3	677
**Bengaluru:**		
Sep 2016-Feb 2017	64.5	31
Mar-Aug 2017	74.7	79
Sep 2017-Feb 2018	70.2	161
Mar-Aug 2018	79.4	214
Sep 2018-Feb 2019	85.8	309
**Hyderabad:**		
Sep 2016-Feb 2017	91.4	162
Mar-Aug 2017	95.0	260
Sep 2017-Feb 2018	94.3	574
Mar-Aug 2018	95.5	422
Sep 2018-Feb 2019	95.9	368
**Male:**		
Sep 2016-Feb 2017	81.5	85
Mar-Aug 2017	87.6	138
Sep 2017-Feb 2018	85.5	343
Mar-Aug 2018	85.5	305
Sep 2018-Feb 2019	87.4	303
**Female:**		
Sep 2016-Feb 2017	94.1	108
Mar-Aug 2017	94.2	201
Sep 2017-Feb 2018	93.0	392
Mar-Aug 2018	95.1	331
Sep 2018-Feb 2019	96.0	374
**Age <15 years:**		
Sep 2016-Feb 2017	100.0	6
Mar-Aug 2017	100.0	19
Sep 2017-Feb 2018	94.7	38
Mar-Aug 2018	94.1	34
Sep 2018-Feb 2019	100.0	22
**Age 15-49 years:**		
Sep 2016-Feb 2017	91.2	147
Mar-Aug 2017	91.1	247
Sep 2017-Feb 2018	89.2	539
Mar-Aug 2018	91.5	483
Sep 2018-Feb 2019	91.5	520
**Age 50+ years:**		
Sep 2016-Feb 2017	70.0	40
Mar-Aug 2017	84.9	73
Sep 2017-Feb 2018	86.7	158
Mar-Aug 2018	83.2	119
Sep 2018-Feb 2019	88.9	135
**Total**	89.9	2580

CHW – community health worker, TB – tuberculosis

*Note: Successful outcome includes cured and treatment completed TB patients. Only patient whose outcome is known are included.

The successful treatment outcomes according to the characteristics of the TB patients and according to the characteristics of follow-up visits for the total cohorts of patients during the reference periods is given in the **Table 4**. The overall successful treatment outcome was higher in Hyderabad at 95% as compared to Bengaluru at 79%. The successful treatment outcome was found to be relatively higher among females, patients who are aged below 15 years, patients who are single, patients who have extra pulmonary TB, patients who were not previously treated for TB, patients whose initial weight was equal to or above a median reference standard – females (38 kg) and males (43kg) [33], patients who did not miss any doses, and patients who received individual counselling on TB awareness, adherence and nutrition or family level counselling. We noticed a higher proportion of successful treatment outcomes among patients who were visited by CHWs, two or more times in the intensive phase of the treatment. For the follow-up visits during the continuation phase and the total visits, we noticed that as the number of visits increased the successful treatment outcomes also increased.

**Table 4:** Successful treatment outcome and the unadjusted odds ratio according to various characteristics of the patient who are referred and followed-up by the CHW*

Characteristics	% of successful outcomes	Unadjusted OR	*P*-value	Number of cases
**Name of district:**				
Bengaluru‡	79.0			794
Hyderabad	94.7	4.79	<0.001	1,786
**Age (years):**				
<15‡	96.6			119
15-49	90.8	0.34	0.038	1,936
50+	85.0	0.20	0.002	525
**Gender:**				
Female‡	94.5			1,174
Male	86.0	0.35	<0.001	1,406
**Marital status:**				
Currently married‡	88.2			1,605
Marriage dissolved	87.1	0.90	0.673	170
Single (Never married)	93.9	2.07	<0.001	805
**HIV status:**				
Negative‡	88.6			1,919
Positive	72.0	0.33	0.014	25
Unknown	94.3	2.14	<0.001	636
**Type of TB:**				
Extra Pulmonary TB‡	94.9			451
Pulmonary TB	88.8	0.43	<0.001	2,129
**History of previous TB treatment:**				
No‡	90.6			2,331
Yes	82.7	0.49	<0.001	249
**Initial weight:†**				
Below median value‡	86.8			310
Median value or above	91.3	1.60	0.027	758
Unknown	89.8	1.34	0.117	1,511
**Patient's relationship with care supporter:**				
No care supporter‡	92.6			512
Parent	90.5	0.77	0.205	728
Siblings/Son/Daughter	85.0	0.45	<0.001	386
Spouse	88.8	0.64	0.029	678
Others	92.8	1.03	0.928	276
**Number of visits during CP:**				
<2‡	57.5			348
2-3	91.0	7.51	<0.001	446
4+	95.9	17.36	<0.001	1,786
**Number of visits during IP:**				
<2‡	82.3			417
2-3	92.2	2.55	<0.001	1,191
4+	90.3	2.02	<0.001	972
**Total number of follow-up visits:**				
<4‡	63.7			311
4-7	91.7	6.29	<0.001	1,022
8+	94.9	10.73	<0.001	1,247
**Missed any doses:**				
No‡	91.2			2,264
Yes	80.7	0.41	<0.001	316
**Provided TB awareness counselling:**				
No‡	88.6			2,089
Yes	95.5	2.75	<0.001	491
**Provided adherence counselling:**				
No‡	87.7			1888
Yes	96.0	3.34	<0.001	692
**Provided nutritional counselling/support:**				
No‡	88.3			1,960
Yes	94.8	2.43	<0.001	620
**Provided family level counselling:**				
No‡	89.5			2,357
Yes	94.2	1.90	0.029	223
Total	89.9			2,580

CHW – community health worker, TB – tuberculosis, HIV – human immunodeficiency virus, IP – intensive phase, CP – continuation phase, OR – odds ratio

*Note: successful outcome includes cured and treatment completed TB patients.

†Considered median value of 43 kg for males and of 38 kg for females above 15 years. For children, we have considered as median or above, if the weight is known.

‡Reference category.

We noticed that a number of patient as well as follow-up visit characteristics were associated with successful treatment outcomes. Hence, we applied multiple logistic regression models in order to identify any significant improvement in the successful treatment outcomes across the referral cohorts when the other potential factors are controlled for. We conducted three separate models while considering the follow-up visits during intensive phase, continuation phase as well as the total visits made, in order to know how controlling for these differs for the successful treatment outcomes across the referral cohorts.

The first model included the number of visits in the intensive phase as well as all other significant factors as per the backward stepwise multiple logistic regression model. This model indicated that the successful treatment outcomes over the different referral cohorts were not significant and thus were not included in the model (**Table 5**). In other words, we did not notice any independent effect of the referral cohorts on the successful treatment outcomes in the model that considered the follow-up visits in the intensive phase as a control variable. The same model also suggested that successful treatment outcomes were significantly higher in Hyderabad than Bengaluru, patients who were never married, who received more than 2 follow-up visits during the IP visits, whose initial weight is more than the median weight according to sex, who received adherence counselling and who received nutritional counselling and/or nutritional support during the follow-up visits. Male patients, patients who have pulmonary TB, who are previously treated for TB and who missed any dose are significantly less likely to experience a successful treatment outcome. Though the initial weight and nutritional counselling and/or nutritional support were identified to be a significant factor in the first model, in the second and third model its effect was not significant and hence this was excluded in the models that included the follow-up visits during the continuation phase and the total follow-up visits, respectively.

**Table 5:** Results of multivariate logistic regression model for successful outcome among TB patient referred and followed-up by CHW

	Model 1*			Model 2*			Model 3*		
**Characteristics**	**AOR**	**95% CI**	***P*-value**	**AOR**	**95% CI**	***P*-value**	**AOR**	**95% CI**	***P*-value**
**Referral cohort:**									
Sep 2016-Feb 2017†									
Mar-Aug 2017				1.11	(0.56-2.20)	0.760	1.16	(0.61-2.23)	0.653
Sep 2017-Feb 2018				0.74	(0.40-1.35)	0.326	0.81	(0.45-1.44)	0.468
Mar-Aug 2018				1.35	(0.72-2.54)	0.346	1.25	(0.68-2.28)	0.477
Sep 2018-Feb 2019				2.48	(1.28-4.81)	0.007	2.21	(1.15-4.27)	0.018
**Name of district:**									
Bengaluru†									
Hyderabad	5.45	(3.75-7.92)	<0.001	5.15	(3.43-7.75)	<0.001	6.39	(4.17-9.78)	<0.001
**Gender:**									
Female†									
Male	0.48	(0.35-0.67)	<0.001	0.44	(0.30-0.62)	<0.001	0.43	(0.30-0.60)	<0.001
**Marital status:**									
Currently married†									
Marriage dissolved	0.88	(0.51-1.51)	0.639				0.89	(0.49-1.61)	0.693
Single	1.68	(1.14-2.48)	0.008				1.59	(1.05-2.40)	0.029
**HIV status**									
Negative†									
Positive				0.28	(0.09-0.85)	0.025	0.30	(0.10-0.88)	0.029
Unknown				1.42	(0.89-2.28)	0.141	1.21	(0.77-1.91)	0.418
**Type of TB**									
Extra Pulmonary TB†									
Pulmonary TB	0.65	(0.41-1.05)	0.076	0.50	(0.30-0.85)	0.01	0.58	(0.35-0.96)	0.034
**History of previous TB treatment:**									
No†									
Yes	0.63	(0.42-0.97)	0.035	0.56	(0.35-0.9)	0.016	0.58	(0.36-0.91)	0.019
**Patients' relationship with care supporter:**									
No care supporter†									
Parent	1.23	(0.76-2.01)	0.398	2.23	(1.31-3.82)	0.003	1.48	(0.88-2.50)	0.139
Siblings/Son/Daughter	0.79	(0.48-1.29)	0.347	1.10	(0.63-1.93)	0.729	0.94	(0.55-1.61)	0.828
Spouse	1.45	(0.89-2.34)	0.133	1.60	(0.95-2.68)	0.076	1.49	(0.89-2.48)	0.126
Others	1.36	(0.76-2.45)	0.297	1.97	(1.03-3.75)	0.041	1.74	(0.93-3.26)	0.082
**Number of visits during CP**									
<2†									
2-3				7.56	(4.86-11.74)	<0.001			
4+				28.89	(19.54-42.72)	<0.001			
**Number of visits during IP**									
<2†									
2-3	2.66	(1.84-3.84)	<0.001						
4+	3.85	(2.61-5.69)	<0.001						
**Total number of follow-up visits**									
<4†									
4-7							6.48	(4.44-9.45)	<0.001
8+							22.71	(14.71-35.06)	<0.001
**Missed any doses:**									
No†									
Yes	0.34	(0.23-0.49)	<0.001	0.23	(0.15-0.35)	<0.001	0.25	(0.16-0.37)	<0.001
**Provided adherence counselling**									
No†									
Yes	2.06	(1.28-3.31)	0.003				1.62	(0.99-2.65)	0.057
**Provided nutritional counselling/support:**									
No†									
Yes	1.88	(1.19-2.98)	0.007						
**Initial weight**									
Below median value†									
Median value or above	1.63	(1.03-2.59)	0.038						
Unknown	1.06	(0.67-1.67)	0.814						

CHW – community health worker, TB – tuberculosis; HIV – human immunodeficiency virus, IP – intensive phase, CP – continuation phase, AOR – adjusted odds ratio, CI – confidence interval

*Model 1 is based on the stepwise backward deletion multivariate logistic regression with 0.10 *P* value as a group as a whole and included the number of follow-up visits during the intensive phase and other characteristics of the patient. Model 2 is based on the stepwise backward deletion multivariate logistic regression with 0.10 *P* value as a group as a whole and included the number of follow-up visits during the continuation phase and other characteristics of the patient. Model 3 is based on the stepwise backward deletion multivariate logistic regression with 0.10 *P* value as a group as whole and included the number of follow-up visits and other characteristics of the patient.

†Reference category.

In the second model, we found significantly higher successful treatment outcome for patients whose care supporter is parent, and other relatives; such as uncle/aunt, in-laws, grand-parents or friends as compared to patients who are under self-care. The result also suggests that the likelihood of successful treatment outcome is significantly higher as the number of follow-up visits during the continuation phase are increased to 2 or more visits. Also, we noticed that HIV positive TB patients were found to have significantly lower successful treatment outcomes.

According to the third model, the chance of successful treatment increases as the total number of follow-up visits is 4 or more as compared to patients who received less than 4 follow-up visits totally. The results from the second and third model indicated that the cohorts of patients who were referred during September 2018 and February 2019 have significantly higher likelihood of completing the treatment successfully as compared to the patients who were referred during September 2016 and February 2017.

The common variables that we identified to have significantly influenced the successful treatment outcome in all the three models were sex of the patient, place (city) of patient, type of TB, status of previous TB treatment and the experience of missed doses. The findings from the analysis also indicate that the CHW approach to improve the TB care cascade may not yield immediate results and may require minimum of two years of implementation to get significant positive results.

## DISCUSSION

The THALI project recruited and trained CHWs to engage with communities for TB detection through the ‘screening pathway’ and for treatment follow-up and support. Findings demonstrated that this is a fairly effective mode of identifying TB within vulnerable communities and ensuring treatment adherence in order to improve TB treatment outcomes. The CHWs require some time to establish credibility within the community and with the local health services. This is evident in that for the initial few months the proportion of referred persons undergoing the TB testing was comparatively lower. Evidence from the analysis indicates that systematic and consistent community outreach activities by the trained CHWs steadily improved TB detection and TB treatment outcomes. Community engagement through CHWs is a slow process, but it plugged gaps in the continuum of care cascade, resulting in improved TB case detection and TB treatment outcomes over time in the urban slums of both metro cities. The results also substantiate the fact that specific sub-populations need more attention to enhance successful treatment outcomes [34]. The reasons for a difference in TB case detection and successful treatment outcomes between the two cities needs further exploration.

A meta-analysis estimated that 39% (95%CI: 30%–49%) of presumptive TB adults had not seen any medical provider [35]. Another study suggested that 46% of the respondents with cough of 2 weeks or more in the districts from the southern India did not seek health care [36]. Through the CHW approach adopted, we were able to identify such individuals, facilitate TB testing and support treatment initiation. The changes in the health seeking can be noted through the increase in the number of referred persons undergoing test, being detected and initiated on TB treatment. This points towards the need for active case finding through the ‘screening pathways’ adopted in urban slums in these two cities [37].

According to the recent TB India report, the percentage of recently diagnosed TB patients put on treatment was 94% in Karnataka and 97% in Telangana, which is lower than the 99% of patients put on treatment from the CHW referred patients in these two cities [38]. The overall successful treatment outcomes rate was 87% in Karnataka (re-calculated after removing the patients who are not evaluated and whose regimen changed) which is close to the rate identified for the latest cohort of TB patients referred in Bengaluru (86%). However, the success rate was 92% in Telangana which is slightly lower than the rate identified for the latest cohorts of TB patients referred and cared for by CHWs in Hyderabad (96%). The CHW approach was able to retain a high proportion of patients on treatment and improve successful TB treatment outcomes as compared to the state average. Additionally, the increase is substantial in Bengaluru and among those aged above 50 years of age.

At the national level, the successful treatment outcomes among the TB patients notified in the years 2016 and 2018 increased slightly from 79% to 81% [38,39]. Similarly, in Telangana state (for the year 2016 the successful rate of Andhra Pradesh is considered for Telangana. Treatment outcome of microbiologically confirmed new TB patients notified in 2016 from public sector is presented. Treatment outcome of TB patients notified in 2018 (Total) is reported here), the successful treatment outcome slightly increased from 89% to 90% over the same period. However, in Karnataka state, the successful treatment outcome remained constant at 80%. The present study indicates a higher increment in the successful treatment outcome than that observed at the national or state level through the consistent follow-up of CHWs, reinforcing the fact that consistent follow-up by the CHW during the TB treatment can improve TB treatment outcomes.

A recently published study from Myanmar reported similar treatment outcomes between cases identified actively and passively [40]. Similarly, another study in India did not find any significant difference in the TB treatment outcomes of people detected through active case finding as compared to passive case finding among marginalized and vulnerable populations [41]. Previous studies had the limitation of not adjusting for the potential baseline characteristics and also the follow-up characteristics [40].

Although we noticed an overall increase in the number of follow-up visits over the time period as observed for the various referral cohorts, Bengaluru witnessed a reduction in the number of follow-up visits. Moreover, the mean number of visits is lower than the expected as per protocol. This could be the result of an additional number of patients who were to be followed up in Bengaluru in addition to the CHW referred patients, as well as a result of higher CHW turnover during the initial years. The program in later stages demanded that CHWs follow up all patients in their area, irrespective of whether they had initially referred them or not. However, this increase in the patient load could have increased the burden on the CHWS, thereby reducing the number of follow-up visits, particularly in Bengaluru.

Significant treatment outcome was noticed for patients who had counselling on treatment adherence and on nutrition or nutrition support offered through the CHWs and also for patients who did not miss any doses. A study from the Cochrane database suggested that educational or counselling interventions may improve completion of treatment for latent tuberculosis [42]. However, there is insufficient research to determine whether the routine provision of food or energy supplements in addition to standard care results in better TB treatment outcomes, or improved quality of life [43]. Another study found that participants without any missed doses had a higher TB treatment completion rate as compared to participants who missed 10% or more of prescribed doses [44].

The study also demonstrates that a patient centered approach may involve differentiating the intensity and package of care for certain categories of patients [45]. These include patients co-infected with HIV and previously TB treated patients [34]. Special attention may have to be given to those without an adult caregiver [45]. Even after controlling for other potential factors, the successful treatment outcome is significantly higher in Hyderabad than Bengaluru, though we provided similar kind of patient centered approach during the treatment. Further analysis is required to understand the reasons for this difference. Nevertheless, we noticed a significant improvement in the successful treatment outcomes between the first and last referral cohorts as indicated by the adjusted odds ratio.

Though we identified advantages in employing trained CHWs in improving the care cascade, the approach has few limitations. For example, the result indicated 30% of TB diagnosed individuals were not registered for PCS, higher in Hyderabad as compared to Bengaluru, although they were initially referred for TB diagnosis by the CHW. It is possible that a segment of the population will never be willing for individualized follow-up by a CHW. Self-perceived stigma and discrimination could be one of the reasons for this [46,47]. The project encouraged such individuals who were unwilling for individualized follow up, to attend patient support group (PSG) meetings. We propose to evaluate the influence of participation in PSG on TB treatment outcomes in a separate study.

Across the various referral cohorts, we identified that the number of referrals, the number of individuals undergoing diagnostic tests and the number of TB patients identified, increased over time. We are not able to specifically identify which program activity contributed to each step in the care cascade. CHWs were involved in a range of activities and we are unable to establish a direct relationship between each activity and its outcome, with the program data that is available. However, we noted during program reviews that the CHW’s rapport with the community and their credibility with the public health services, increased with time. Another study conducted in Bengaluru and Hyderabad identified that knowledge and health seeking behavior increased within the targeted slum communities [48].

From this study, we are unable to examine changes in the TB related stigma, as a result of CHW interventions. We have reported that high levels of community level TB related stigma persisted even at the end of the project, with women concerned about family’s response, while men’s concern centered around community reactions. The CHWs focused on reducing self-perceived stigma and on eliciting family level support. We have recommended further work to reduce community level TB related stigma [49,50].

Another limitation is that we considered many of the socio-economic, demographic, clinical, treatment characteristics, counselling aspects and follow-up visits, but did not consider factors such as the quality of public health care services, private sector care or societal and environmental aspects in the analysis. These factors could have also influenced outcomes in the care cascade studied. We believe that the ultimate aim of the TB control efforts should be TB detection, timely treatment initiation and completion. The CHW approach has augmented this care cascade within the urban slums in the two cities. Our findings indicate that ideally the follow up must be initiated in the IP of treatment, and must be continued into the CP. In fact, the development of rapport with TB symptomatics and their family members must begin even before TB detection. We notice that 2 or more visits in the IP and 4 and more visits during CP follow up are associated with successful treatment outcomes of more than 90%. It is possible that death rates are higher in the intensive phase, as a result of which later follow up is not possible. A study conducted in Northwest Ethiopia indicated 57% of TB deaths occurred during the intensive phase of the treatment, and the median time of death was two months since initiation of the treatment [51]. Initiation of follow-up early in TB treatment could not only enhance TB treatment outcomes, but could potentially increase accuracy of TB death reports and reduce the proportion of not-evaluated.

There are a number of challenges in the study. The proportion of children less than 15 years of age detected with TB is 4.9% of all TB cases detected by CHWs. This is lower than the national average of 6.3% [38]. Most parents tend to opt for private sector services for their children’s health issues. Our CHW program referrals were limited to the public sector, as we were not able to engage with the private sector for various reasons. However, we notice that the ratio of females to males detected with TB is much higher than national and state averages [38]. The CHW approach may therefore be useful to detect TB among vulnerable women in the community, but may need to be boosted to ensure that men with TB are not missed. Though we adopted a systematic and consistent patient centered approach we found fluctuations in the care cascade. For example, though the testing rate improved over time, we noted that all the persons referred were not tested, all the persons initiated on treatment did not agree for PCS follow-up visits, and the treatment outcomes are not available for all the patients followed-up. The quality of referrals and quality of interactions with the community and or patient may be important to motivate and change behavior [52,53]. This is also dependent on the patient load and the area covered by the CHWs. We were not able to examine the influence of these aspects on treatment outcome, as we did not have this data. Finally, we did not include the health system level factors including availability, accessibility, facility readiness and quality of TB diagnostic, treatment and care services, which could have influenced the difference observed between Hyderabad and Bengaluru.

## CONCLUSIONS

It is evident from the study that community engagement with slum population conducted through CHW is a feasible approach and augmented the care cascade of TB detection, treatment initiation and successful treatment outcomes over the period. Case detection increased fairly rapidly, but it takes time before treatment outcomes can be improved. It would be useful to estimate the costs of the model for TB patient detection and successful treatment outcomes.
